# Supplementary material for: N, S Dual-Doped Carbon Derived from Dye Sludge by Using Polymeric Flocculant as Soft Template
Source: Nanomaterials (Basel). 2019 Jul 9;9(7):991. doi: 10.3390/nano9070991 (PMC6669867; doi:10.3390/nano9070991)
Supplement: Supplementary file 1 [file nanomaterials-09-00991-s001.pdf]

Supplementary Information

Article

N, S dual-doped carbon derived from dye sludge by using polymeric flocculant as soft template

Daofeng Luan [1,†], Liang Wu [1,†], Tingting Wei [1], Liu Liu [1], Yin Lv [1], Feng Yu [1], Long Chen [1,*], Yulin Shi [1,*]

1 Key Laboratory for Green Processing of Chemical Engineering of Xinjiang Bingtuan, School of Chemistry and Chemical Engineering, Shihezi University, Shihezi 832003, P.R. China. ldf01shzu@163.com (D.L.); wuliang@daxinpharm.com (L.W.); weitingting99@126.com (T.W.); liuliu66shzu@163.com (L.L.); ag_125@163.com (Y.L.); yufeng923@hotmail.com (F.Y.); chenlong2012@sinano.ac.cn (L.C.); shiyulin521@126.com (Y.S.)

* Correspondence: shiyulin@shzu.edu.cn (Y.S.); Tel.: +86-993-2055030
chenlong2012@sinano.ac.cn (L.C.); Tel.: +86-993-2057277

† These two authors contributed equally to this work.

Table S1. BET surface area and pore structure characterization parameters of N, S-DF-x (x=1, 2, 3).

Samples	S_{BET} (m^2g^{-1})	S_{mic} (m^2g^{-1})	S_{mes} (m^2g^{-1})	D_{mic} (nm)	D_{BJH} (nm)	V_{Total} (cm^3g^{-1})	V_{mic} (cm^3g^{-1})	pore volume(%)	
								V<2nm	V>2nm
N, S-DF-1	515.62	471.63	43.99	0.48	4.15	0.26	0.19	65.51%	34.49%
N, S-DF-2	801.14	629.36	171.78	0.42	4.61	0.59	0.31	52.30%	47.70%
N, S-DF-3	380.22	187.46	192.76	0.57	7.17	0.36	0.13	36.11%	63.89%

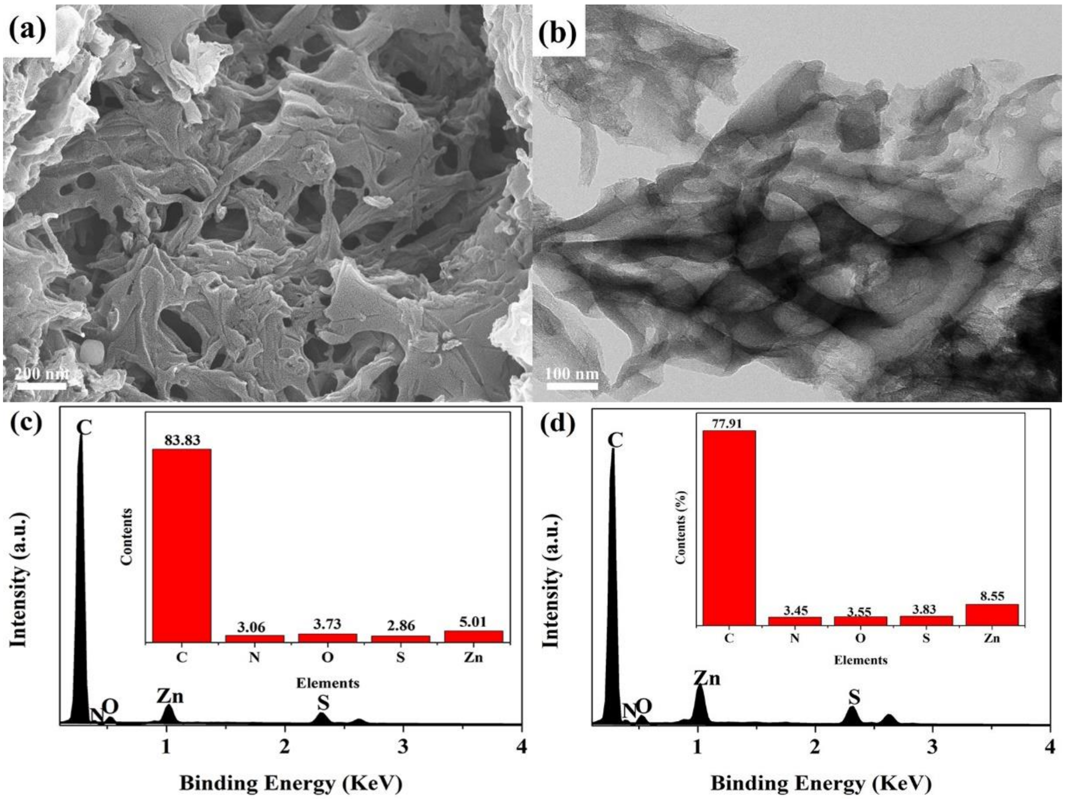

Figure S1. SEM (a) and TEM (b) images of N, S-DF-3; EDS spectrum of N, S-DF-1 (c) and N, S-DF-3 (d).

Table S2. XPS for elemental analyses of N, S-DF-x (x=1, 2, 3) at 800°C carbonization temperature.

Samples	C (at%)	N (at%)	S (at%)
N, S-DF-1	81.67	5.58	2.81
N, S-DF-2	70.16	9.02	4.23
N, S-DF-3	86.78	5.64	1.47

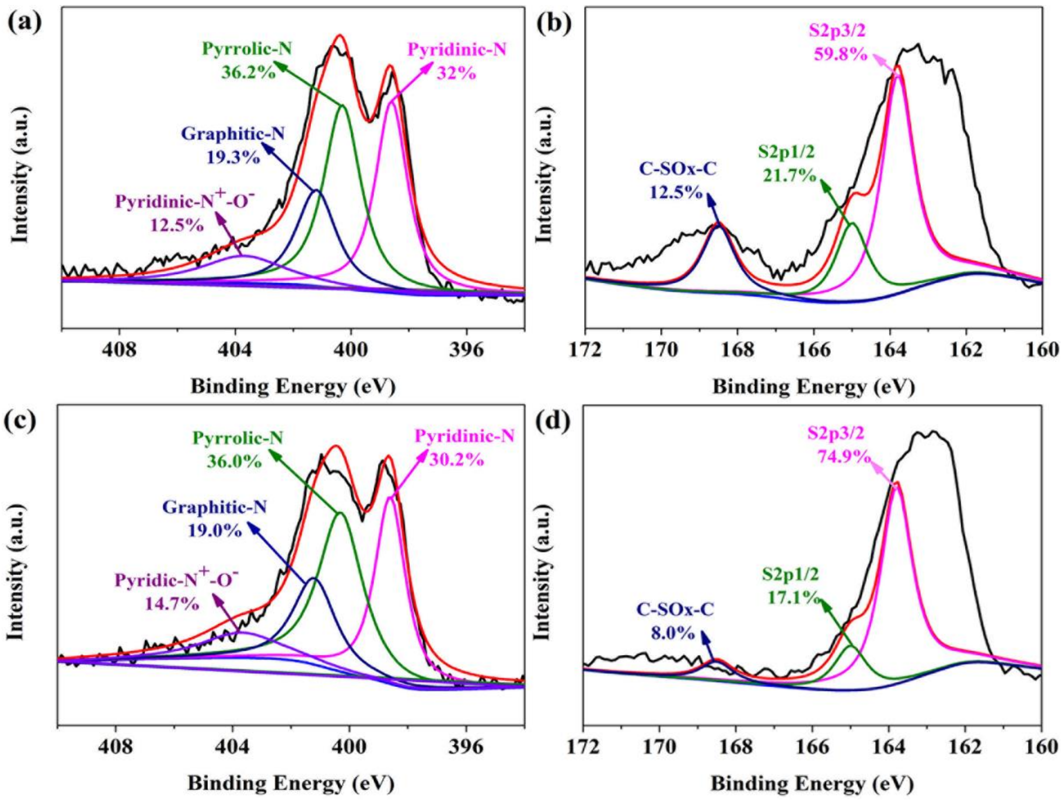

Figure S2. High-resolution XPS deconvoluted spectra of (a) N1s and (b) S2p peak of N, S-DF-1; (c) N1s and (d) S2p peak of N, S-DF-3.

Table S3. The relative ratios of nitrogen species to the total N1s and sulfer species to the total S2p.

Samples	Pyridinic-N	Pyrrolic-N	Graphitic-N	Pyridinic-N^+-O^-	S $2p_{3/2}$	S $2p_{1/2}$	C-SO_x-C
N, S-DF-1	32.0%	36.2%	19.3%	12.5%	59.8%	21.7%	13.5%
N, S-DF-2	36.0%	38.8%	19.7%	4.5%	8.0%	4.0%	88.0%
N, S-DF-3	30.2%	36.0%	19.0%	14.7%	74.9%	17.1%	8.0%

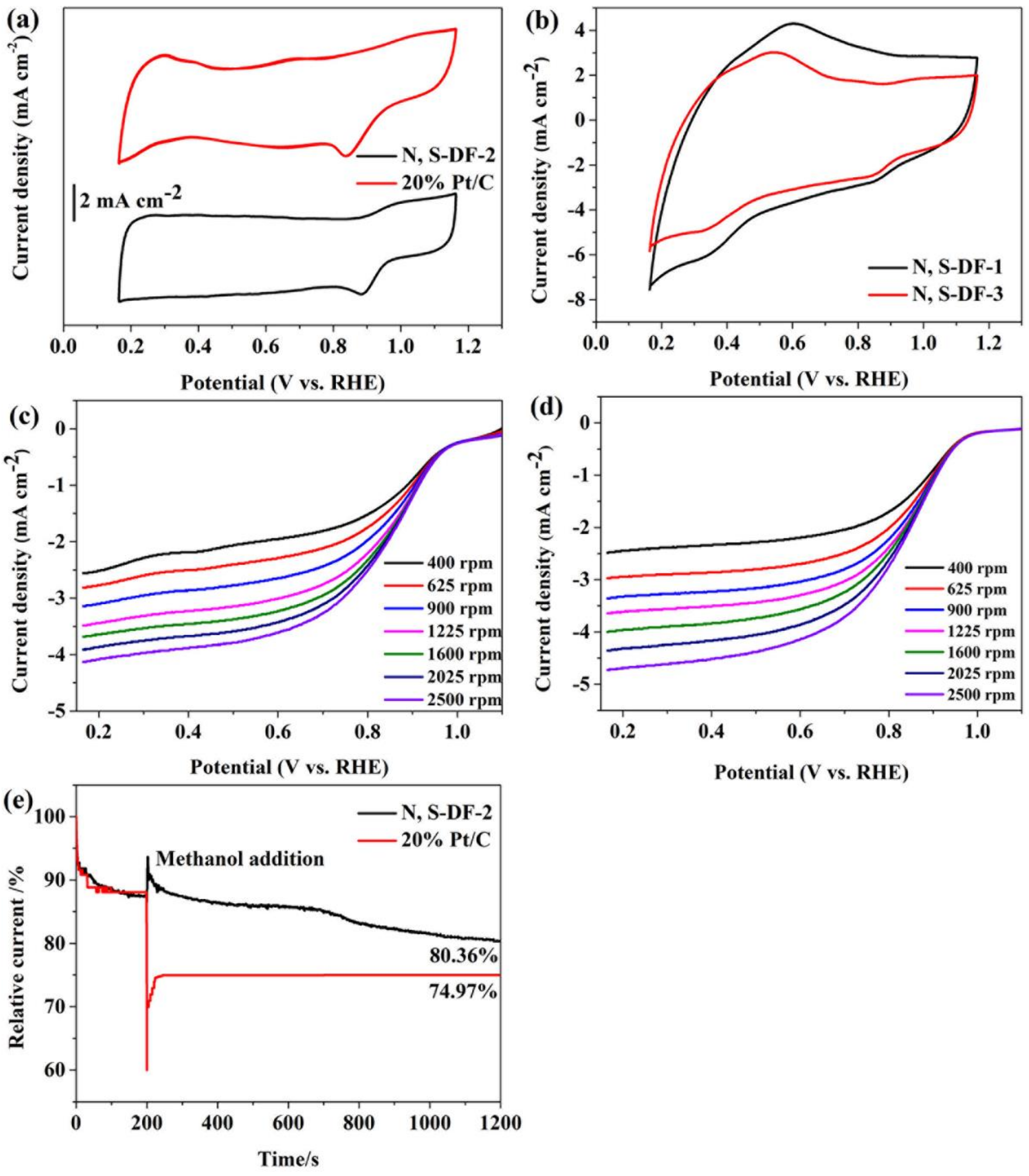

Figure S3. (a) CV curves of N, S-DF-2 and 20% Pt/C in O_2-saturated 0.1 M KOH solutions. (b) CV curves of the N, S-DF-x (x=1, 3) in O_2-saturated 0.1 M KOH solutions with a scan rate of 50 mV s^{-1}. Rotating-disk voltammograms of (c) N, S-DF-1; (d) N, S-DF-3; (e) N, S-DF-2 with the addition of 2.5 mL methanol.

Table S4. A comparison of the nitrogen content and specific capacitance of nitrogen doping carbon materials from the literature.

**Sample**	Nitrogen content (at%)	Specific capacitance ($F\ g^{-1}$) at $1\ A\ g^{-1}$	Refs.
N-doped carbon	9.91	221	[1]
N-doped inverse opal carbon materials	14.72	222	[2]
Nitrogen-doped	8.84	215	[3]
g-CN/NCS-2	38	259	[4]
N-doped hollow carbon spheres	6.68	170	[5]
N, S-DF-2	9.02	230.5	This work

References

1. Cheng, M.; Meng, Y.; Meng, Q.; Mao, L.; Zhang, M.; Amin, K.; Ahmad, A.; Wu, S.; Wei, Z. A Hierarchical Porous N-Doped Carbon Electrode with Superior Rate Performance and Cycling Stability for Flexible Supercapacitors. *Mater. Chem. Front.* **2018**, 2, 986-992.
2. Chen, L.-F.; Huang, Z.-H.; Liang, H.-W.; Gao, H.-L.; Yu, S.-H. Three-Dimensional Heteroatom-Doped Carbon Nanofiber Networks Derived from Bacterial Cellulose for Supercapacitors. *Adv. Funct. Mater.* **2014**, 24, 5104-5111.
3. Jiang, J.; Bao, L.; Qiang, Y.; Xiong, Y.; Chen, J.; Guan, S.; Chen, J. Sol-Gel Process-Derived Rich Nitrogen-Doped Porous Carbon through Koh Activation for Supercapacitors. *Electrochim. Acta* **2015**, 158, 229-236.
4. Zhu, J.; Kong, L.; Shen, X.; Zhou, H.; Zhu, G.; Ji, Z.; Xu, K.; Shah, S.A. Nitrogen-Enriched Carbon Spheres Coupled with Graphitic Carbon Nitride Nanosheets for High Performance Supercapacitors. *Dalton Trans.* **2018**, 47, 9724-9732.
5. Hu, L.; Sun, Y.; Zhang, F.; Chen, Q. Facile Synthesis of Porous Mn2o3 Hierarchical Microspheres for Lithium Battery Anode with Improved Lithium Storage Properties. *J. Alloys Compd.* **2013**, 576, 86-92.
